# Eficacia de la fotobiomodulación en el manejo del dolor agudo en trastornos temporomandibulares: Estudio de serie de casos

**DOI:** 10.21142/2523-2754-1302-2025-245

**Published:** 2025-05-16

**Authors:** Anthony Villafañe, Adalsa Hernández-Andara, Alfredo Vargas, Aubert Brito, Carlos Manresa, Mariana Villarroel-Dorrego

**Affiliations:** 1 Servicio de Cirugía Maxilofacial, Hospital General del Oeste Dr. José Gregorio Hernández. Caracas, Venezuela. anthonymvillafanea@gmail.com, odalfredovargas@gmail.com, aubertbrito@gmail.com, manresa723@gmail.com, mariana.villarroel@ucv.ve Universidad Dr. José Gregorio Hernández Servicio de Cirugía Maxilofacial Hospital General del Oeste Dr. José Gregorio Hernández Caracas Venezuela anthonymvillafanea@gmail.com odalfredovargas@gmail.com aubertbrito@gmail.com manresa723@gmail.com mariana.villarroel@ucv.ve; 2 Centro Diagnóstico Docente Las Mercedes. Caracas, Venezuela. adalsah@gmail.com Centro Diagnóstico Docente Las Mercedes Caracas Venezuela adalsah@gmail.com

**Keywords:** trastorno temporomandibular, dolor orofacial, laserterapia, fotobiomodulación, láser diodo, temporomandibular disorder, orofacial pain, láser therapy, photobiomodulation, diode láser

## Abstract

**Introducción:**

La fotobiomodulación con láser diodo se ha aplicado como una técnica no invasiva para tratar los síntomas de la articulación temporomandibular (ATM), especialmente en el alivio de condiciones dolorosas agudas.

**Objetivo:**

Evaluar el efecto de la fotobiomodulación en el manejo del dolor agudo y la apertura bucal en pacientes con trastorno de la ATM.

**Metodología:**

Fueron incluidos 12 pacientes diagnosticados según los criterios de diagnóstico para los trastornos temporomandibulares (DC/TMD) para aplicaciones clínicas y de investigación de la Red del Consorcio Internacional RDC/TMD de la International Association for Dental Research y el Grupo de Interés Especial en Dolor Orofacial de la International Association for the Study of Pain, tras la firma del consentimiento informado. Todos los pacientes fueron irradiados usando un láser diodo dual Gemini EVO Ultradent, y se usó un protocolo de irradiación extrabucal de 0.2W sobre la ATM y 1W cuando el paciente refería dolor muscular sobre la zona sintomática, con un total de 6 sesiones. La intensidad del dolor se calificó usando una escala numérica (EVA). La función mandibular se evaluó mediante la máxima apertura mandibular sin asistencia medida en mm. Los datos fueron analizados mediante un análisis de varianza de modelo mixto (ANOVA). Los valores p < 0,05 fueron considerados estadísticamente significativos.

**Resultados:**

La intensidad de dolor disminuyó estadísticamente de 7,52 ± 1,18 a 2,13 ± 1,33 (p < 0,001) de la primera a la última sesión. A nivel de región de ATM hubo una reducción de dolor de 6,25 ± 2,83 a 1,5 ± 1,50; en el músculo temporal, de 7 ± 1,85 a 1,66 ± 1,30; en el pterigoideo, de 6,25 ± 2,99 a 1,25 ± 1,15; y en el masetero, de 6,83 ± 1,80 a 1,41 ± 1,31. Con respecto a la apertura bucal, la función aumentó estadísticamente de 32,05 ± 4,3 mm a 42,66 ± 1,6 mm (p < 0,001).

**Conclusiones:**

La intensidad del dolor y la apertura bucal en pacientes con trastorno de la ATM mejoran sustancialmente después de los tratamientos de fotobiomodulación láser.

## SUMMARY

**Introduction:**

Diode láser photobiomodulation has been applied as a non-invasive technique to treat temporomandibular joint (TMJ) symptoms, especially in the relief of acute painful conditions.

**Objective:**

To evaluate the effect of photobiomodulation in the management of acute pain and mouth opening in patients with TMJ disorder.

**Methodology:**

12 patients diagnosed according to the “Diagnostic Criteria for Temporomandibular Disorders (DC/TMD) for Clinical and Research Applications of the International RDC/TMD Consortium Network of the International Association for Dental Research and the Special Interest Group were included. in orofacial pain from the International Association for the Study of Pain”, after signing the informed consent. All patients were irradiated using Gemini EVO Ultradent dual diode láser, using an extraoral irradiation protocol of 0.2W over the TMJ and 1W when the patient reported muscle pain over the symptomatic area, with a total of 6 sessions. Pain intensity was rated using a numerical scale (VAS). Mandibular function was evaluated by the maximum unassisted mandibular opening measured in mm. Data were analyzed using a mixed model analysis of variance (ANOVA). Values p<0.05 were considered statistically significant.

**Results:**

Pain intensity statistically decreased from 7.52±1.18 to 2.13±1.33 (p<0.001) from the first to the last session. TMJ region showed a reduction in pain from 6.25 ± 2.83 to 1.5 ± 1.50, in the temporalis muscle from 7 ± 1.85 to 1.66 ± 1.30, in the pterygoid from 6.25 ± 2.99 to 1.25 ± 1.15 and, in the masseter 6.83 ± 1.80 to 1.41 ± 1.31. Regarding mouth opening, the function statistically increased from 32.05±4.3mm to 42.66±1.6mm (p<0.001).

**Conclusions:**

Pain intensity and mouth opening in patients with TMJ disorder improve substantially after láser photobiomodulation treatments.

## INTRODUCCIÓN

La Academia Americana de Dolor Orofacial define los trastornos temporomandibulares (TTM) como un término colectivo que incluye un grupo de condiciones musculoesquelético y neuromusculares que involucran los músculos masticatorios, la articulación temporomandibular (ATM) y las estructuras asociadas^1^. El dolor que limita la apertura de la boca, los movimientos asimétricos de la mandíbula y los sonidos de la ATM son los hallazgos más comunes en los TTM. A su vez, estos síntomas comprometen la calidad de vida, el sueño y el bienestar psicológico, lo que genera ansiedad, estrés, depresión y un efecto negativo en la función social, la salud emocional y el nivel de energía. Se estima que más del 40% de la población muestra signos y síntomas asociados a la ATM, y ocurren con más frecuencia entre las mujeres que entre los hombres ^1^.

La etiología de los TTM es variable y controvertida. Ahora se consideran enfermedades multifactoriales que incluyen anomalías posturales, parafunciones oclusales y factores psicológicos y sociales, que actúan sinérgicamente en el inicio y el curso de la enfermedad ^2^.

Debido a que los TTM representan un grupo de trastornos variados, con diversas etiologías, existen tratamientos específicos para cada uno. El objetivo principal de muchos tratamientos es reducir la hiperactividad muscular, lo que genera la relajación muscular, restauración de la actividad normal de la ATM y toda la región, así como la reducción del dolor, el espasmo y el edema ^3^. Estos tratamientos se basan en un enfoque multidisciplinario que incluye modalidades de farmacoterapia ^4^, fisioterapia como la terapia manual ^5^, electroterapia ^6^, ultrasonido ^7^, electroterapia transcutánea estimulación nerviosa (TENS) y fotobiomodulación ^8^. 

La terapia con láser de baja intensidad o potencia es un enfoque novedoso, no invasivo y rentable en el campo de la salud. Debido a sus propiedades únicas, la irradiación láser de baja potencia puede promover el metabolismo celular, reducir el dolor, mejorar el proceso de cicatrización de heridas (efecto regenerador/reparador), reducir el edema y acelerar el proceso de inflamación. La fotobiomodulación se ha empleado como una modalidad de tratamiento para una variedad de condiciones en medicina y odontología, incluido el síndrome de dolor musculoesquelético, lesiones y ulceraciones de tejidos blandos, hipersensibilidad de la dentina y atenuación de las complicaciones de los procedimientos quirúrgicos ^8^^-^^15^.

La fotobiomodulación consiste en un tratamiento que puede promover modificaciones celulares y tisulares inducidas por una mayor actividad tanto de las mitocondrias como de la bomba sodio/potasio, lo que genera un aumento de la vascularización y el crecimiento de fibroblastos. Estos cambios dan como resultado procesos de cicatrización mejorados y una reducción notoria del dolor. En los últimos años se han demostrado las propiedades terapéuticas del láser diodo en la analgesia del dolor agudo y crónico ^14^.

Los efectos de la fotobiomodulación sobre los TTM incluyen efectos inflamatorios y analgésicos. Poco después de su aplicación, los láseres de baja intensidad han demostrado su capacidad para ayudar en el tratamiento de los síntomas del dolor. Una ventaja significativa de la terapia con láser para los TTM es que se trata de una terapia no invasiva, lo que reduce la necesidad de cirugía o el uso de medicamentos para aliviar el dolor y regenerar los tejidos ^11^^,^^12^. La terapia con láser ha demostrado aliviar el dolor de los pacientes con TTM minutos después de su aplicación, lo que permite al paciente retomar sus actividades y mejora así su calidad de vida ^10^^,^^12^^,^^15^.

Nadershah *et al*. ^11^ evaluaron el dolor a nivel de la ATM y los músculos de la masticación durante la función usando una longitud de onda de 940 nm. Se concluyó que la terapia de fotobiomodulación es eficaz a corto plazo para aliviar el dolor miofacial por TTM, no es invasiva, resulta fácil de aplicar y no tiene efectos secundarios sistémicos. Interesantemente, Del Vecchio *et al*. ^12^ mostraron un efecto superior de la fotobiomodulación sobre la terapia farmacológica en el manejo del dolor en TTM.

Sobral *et al*. ^9^ y Xu *et al*. ^10^, mediante estudios de metaanálisis, concluyeron que la fotobiomodulación generaba un alivio a la sintomatología dolorosa, aunque los estudios revisados tenían una calidad de evidencia moderada. Por tanto, sugirieron que las investigaciones futuras deberían definir cuidadosamente la población de estudio y proporcionar la justificación de los parámetros elegidos. Esto facilitaría no solo la replicación en el entorno clínico, sino que también mejoraría la homogeneidad de los ensayos y permitiría agrupar los datos. Además, es necesario examinar diferentes parámetros del láser, regímenes de tratamiento, tiempos de evaluación y medidas de resultados porque no es invasivo, es seguro, fácil de usar y económico.

Por tal razón, el objetivo del presente estudio fue evaluar la eficacia de la fotobiomodulación en el manejo del dolor agudo en pacientes con TTM.

## METODOLOGÍA

Esta investigación cumplió con las normas éticas que sirven para promover el respeto a todos los seres humanos y protegen la salud y sus derechos individuales, enmarcadas en la Declaración de Helsinki de la Asociación Médica Mundial. Esta investigación cumplió con los principios bioéticos (beneficencia, no maleficencia, justicia y autonomía). Los datos aquí presentados fueron tomados a partir de los pacientes que están incluidos en este estudio sin comprometer su confidencialidad; a su vez, se presentó un consentimiento informado aprobado por el Comité de Bioética del Hospital General del Oeste (HGO-BIOÉTICA-9/2023).

Fueron incluidos en el presente reporte 12 pacientes que asistieron al servicio de Cirugía Maxilofacial del Hospital General del Oeste Dr. José Gregorio Hernández con dolor agudo a nivel de la ATM y/o los músculos masticatorios y tejidos relacionados.

Fueron considerados los siguientes criterios para la selección de los pacientes:

- Dolor en el área de la ATM y/o irradiado a nivel facial, mandibular o cervical, en los últimos 30 días, no relacionado con otras condiciones no asociadas a la articulación como cuadros infecciosos, neuralgias, etc.
- Limitación de la apertura oral.
- Chasquidos o clics dolorosos en la apertura y cierre bucal.
- Sensibilidad a la palpación en región de ATM y/o músculos: temporales, maseteros y pterigoideos.

Asimismo, fueron excluidos pacientes que hubieran recibido terapia analgésica antes del inicio del tratamiento o para quienes se contraindicara el tratamiento láser por alguna razón (como el embarazo). 

### Evaluación clínica

Todos los pacientes fueron evaluados siguiendo los criterios de diagnóstico para los TTM (DC/TMD) para aplicaciones clínicas y de investigación de la Red del Consorcio Internacional RDC/TMD de la International Association for Dental Research (actualmente denominada INFORM, acrónimo de International Network for Orofacial Pain and Related Disorders Methodology) y el Grupo de Interés Especial en Dolor Orofacial de la International Association for the Study of Pain ^16^, en el cual se valoran dos ejes fundamentales: el eje I, que incluye los aspectos relacionados con el dolor, datos demográficos y el algoritmo para la clasificación del TTM; y el eje II, que contiene los aspectos psicosociales, conductuales y emocionales del paciente.

### Evaluación imagenológica

A los pacientes se les realizó una ortopantomografía para excluir dolencia odontogénica, así como una resonancia magnética para observar alteraciones de tejido blando a nivel articular y tomografía computarizada, con el fin de evaluar componentes óseos de la ATM que pueden estar afectados. 

### Variables cuantificadas

#### Dolor

El dolor fue cuantificado mediante la Escala Visual Analógica (EVA), que permite medir la intensidad del dolor que experimenta y describe el paciente con la máxima reproducibilidad entre los observadores. Esta consiste en una línea horizontal de 10 centímetros en cuyos límites se encuentran las expresiones extremas de un síntoma. El dolor fue medido en la ATM y en la región de los músculos: temporal, pterigoideo y masetero. 

#### Apertura bucal

**Figura 1:**
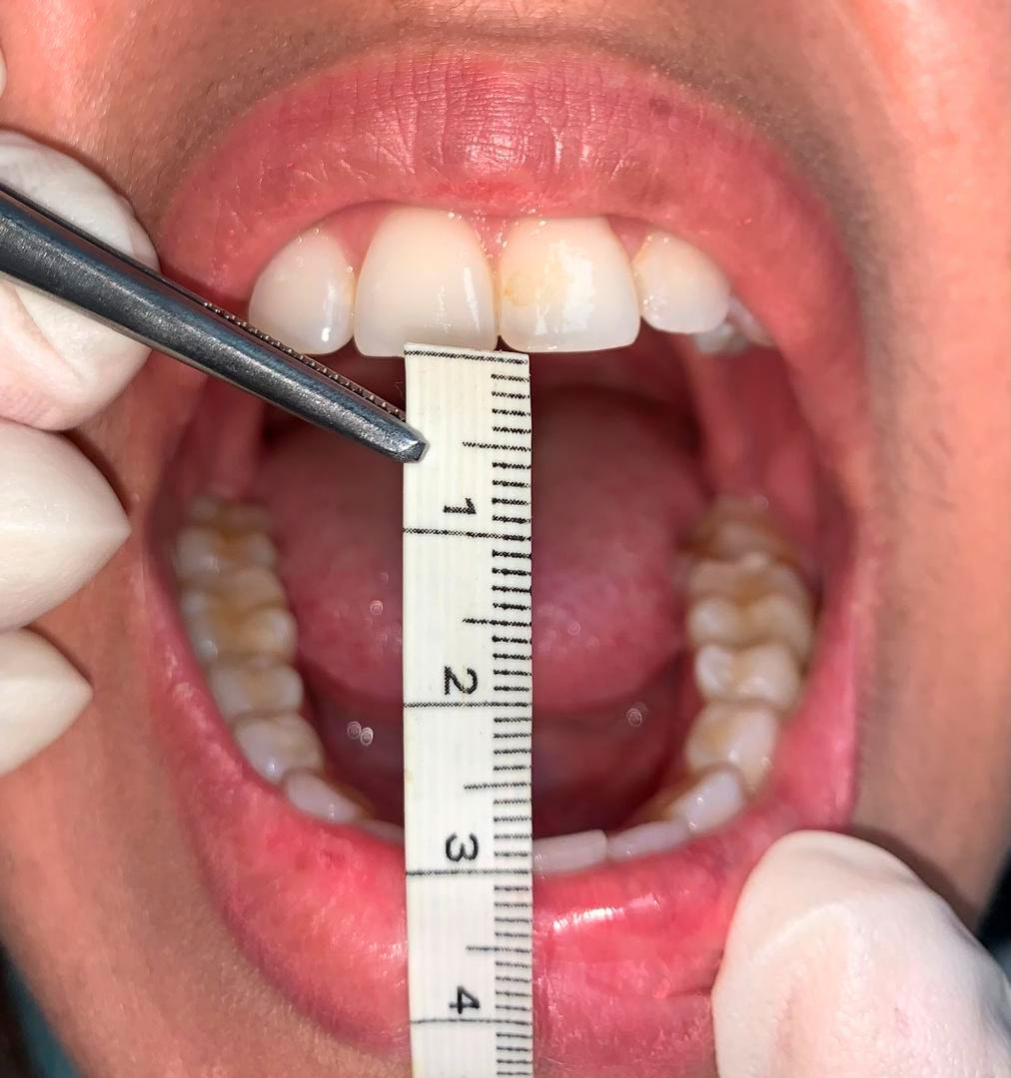
Evaluación de la apertura bucal mediante escala de medición en milímetros (mm)

La apertura bucal fue calculada en valores numéricos en milímetros usando una cinta métrica. Se indicó al paciente que abriera la boca poco a poco hasta el momento de dolor. En este punto, se midió la distancia entre los bordes incisivos de los dientes anteriores maxilares y mandibulares (Figura 1). 

### Terapia láser

El tratamiento de fotobiomodulación para los pacientes consistió en la irradiación con láser diodo Gemini EVO^TM^ Ultradent de longitud de onda dual 810/980 nm, haz superpulsado a una potencia de 0,2 W y punta de 7 mm para el área articular, así como una potencia de 1,0 W y punta de 25 mm para las áreas temporal, masetero y pterigoideo. El tiempo de irradiación para cada zona fue de 60 segundos y la fluencia fue de 31,57 J/cm^2^ en área muscular y 12,21 J/cm^2^ en área articular. El protocolo adoptado consistió en dos tratamientos semanales durante seis semanas. 

### Análisis estadístico

Los datos fueron analizados mediante un análisis de varianza de modelo mixto (ANOVA). Los valores p < 0,05 fueron considerados estadísticamente significativos.

## RESULTADOS

### Género y edad

La muestra estuvo conformada por 11 mujeres (91,67%) y 1 hombre (8,33%), con una media de edad de 38,33 ± 12,39 años.

### Dolor

La evaluación del dolor general se realizó mediante la media ± desviación estándar de los valores de ATM y músculos por sesión (Tabla 1). Se observó una reducción estadísticamente significativa del dolor desde la segunda sesión (7,5 ± 1,8 a 2,13 ± 1,33) (p < 0,001). Es interesante anotar que, en la tercera sesión, se observó un ligero aumento del dolor; sin embargo, siempre menor que la medición inicial. 

**Figura 2:**
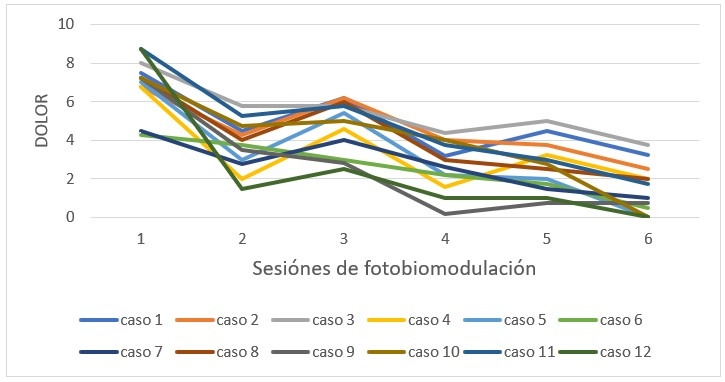
Media de dolor general por paciente

Al evaluar el dolor de forma individual, su disminución general fue observada en cada paciente de forma independiente, en un mismo patrón de descenso (Figura 2).

**Tabla 1:** Comparación de media de dolor general (ATM y regiones musculares evaluadas)

Sesión	Media	Desviación estándar	p*
1	7,52	1,18	> 0,001
2	3,41	1,54	
3	4,75	2,04	
4	2,54	1,45	
5	2,64	1,39	
6	2,13	1,33	

*Análisis realizado mediante prueba ANOVA (análisis de varianza) de modelo mixto.

### Dolor ATM

Al evaluar el dolor en la ATM (Tabla 2), se observó una disminución estadísticamente significativa desde la segunda sesión (6,25 ± 2,83 a 1,50 ± 1,50) (p < 0,001). De nuevo, en la tercera sesión se observó un leve aumento del dolor, pero de menor medida en comparación con la medición inicial.

**Figura 3:**
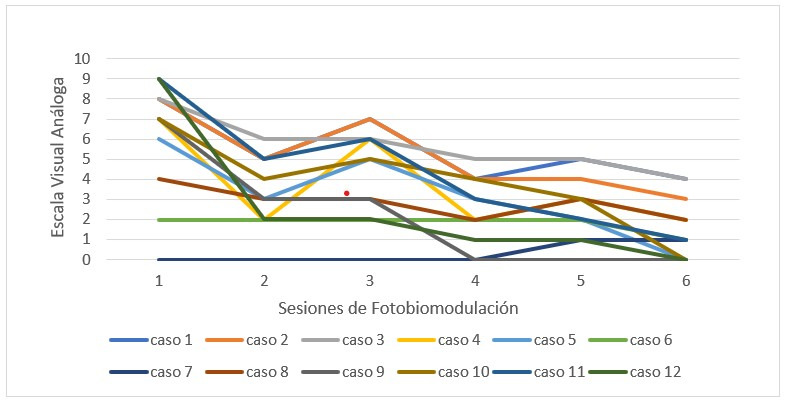
Dolor en área de ATM en cada paciente

Al evaluar el dolor de cada individuo, se observó una disminución independiente en cada uno, en un modelo semejante (Figura 3).

**Tabla 2:** Dolor en área de ATM

Sesión	Media	Desviación estándar	p*
1	6,25	2,83	> 0,001
2	3,33	1,72	
3	4,33	2,26	
4	2,50	1,62	
5	2,58	1,56	
6	1,50	1,50	

*Análisis realizado mediante prueba ANOVA (análisis de varianza) de modelo mixto.

### Dolor muscular

#### Dolor en área de músculo temporal

El músculo temporal fue la región más afectada, cuando se le comparó con los otros músculos. La disminución del dolor, que inició en 7 ± 1,85, descendió a 1,66 ± 1,30 (p < 0,001) (Tabla 3).

**Tabla:** 3. Dolor en área del músculo temporal

Sesión	Media	Desviación estándar	p*
1	7	1,85	> 0,001
2	3,50	1,44	
3	5,16	1,85	
4	2,58	1,37	
5	2,83	1,26	
6	1,66	1,30	

*Análisis realizado mediante prueba ANOVA (análisis de varianza) de modelo mixto.

**Figura 4:**
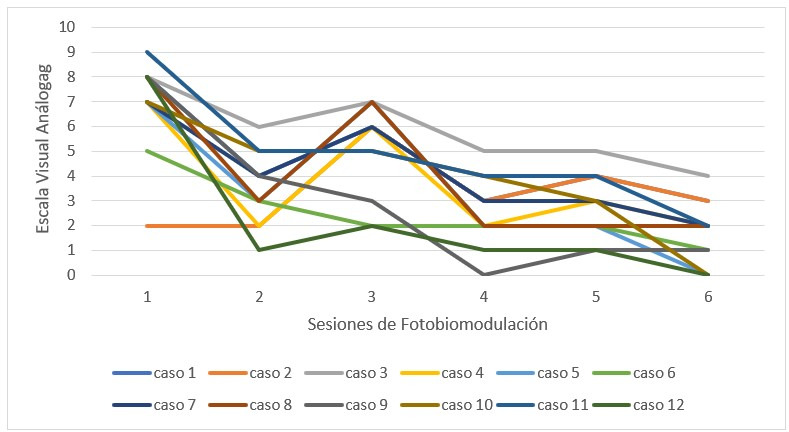
Dolor en área del músculo temporal

Cuando el dolor se evaluó de manera individual (Figura 4), cada paciente experimentó una disminución del dolor en un patrón ya descrito anteriormente.

#### Dolor en área de músculo pterigoideo

Cuando fue valorada el área de músculo pterigoideo, se apreció, de nuevo, una reducción estadísticamente significativa del dolor (de 6,25 ± 2,99 a 1,25 ± 1,15) (p < 0,001).

**Figura 5:**
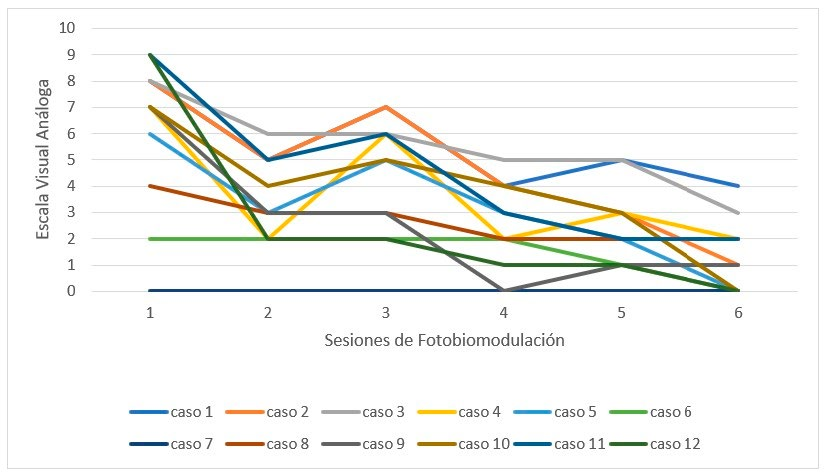
Dolor en área del músculo pterigoideo

Al observar el dolor de cada persona, se apreció en cada sujeto un descenso del dolor en el mismo patrón descrito. Un paciente refirió no sentir dolor desde el inicio (Figura 5).

**Tabla 4:** Dolor en área del músculo pterigoideo

SESIÓN	Media	Desviación estándar	p*
1	6,25	2,99	0,0001
2	3,33	1,69	
3	4,33	2,09	
4	2,5	1,61	
5	2,33	1,41	
6	1,25	1,15	

*Análisis realizado mediante prueba ANOVA (análisis de varianza) de modelo mixto.

#### Dolor en área de músculo masetero

El músculo masetero fue la segunda región muscular más dolorosa. Su respuesta ante la irradiación láser fue similar a las otras regiones musculares, y mostró una disminución estadísticamente significativa del dolor desde la segunda sesión (6,83 ± 1,80 a 1,41 ± 1,31) (p < 0,001) (Tabla 5). 

**Tabla 5:** Comparación de dolor en área del músculo masetero

Sesión	Media	Desviación estándar	p*
1	6,83	1,80	> 0,001
2	3,75	1,54	
3	5,08	1,83	
4	3	1,70	
5	2,83	1,33	
6	1,41	1,31	

*Análisis realizado mediante prueba ANOVA (análisis de varianza) de modelo mixto.

**Figura 6:**
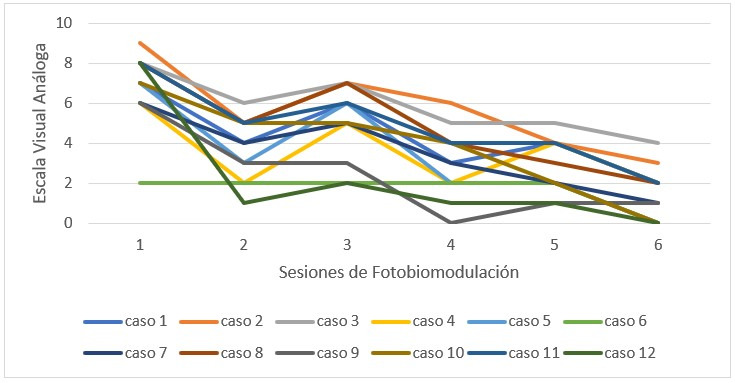
Dolor en área del músculo masetero

Al igual que las otras regiones, todos los pacientes mostraron un discreto aumento del dolor en la tercera sesión, que no superó nunca el valor inicial de dolor (Figura 6).

### Apertura bucal

**Figura 7:**
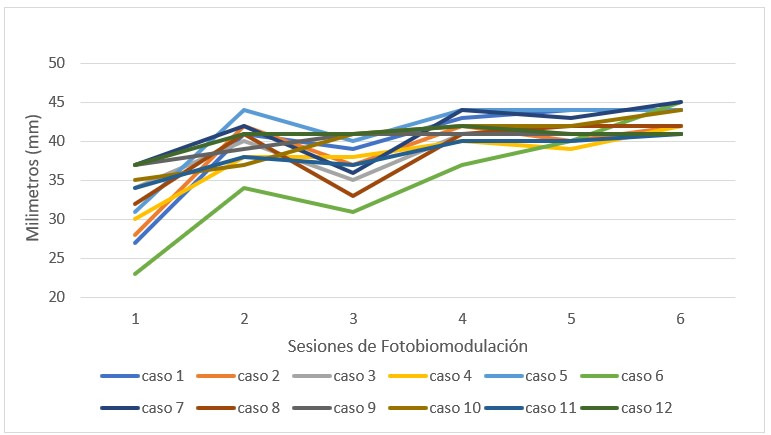
Apertura oral medida en escala métrica en milímetros (mm)

La apertura bucal mostró un drástico ascenso posterior entre la primera sesión y la última (32,08 ± 4,38 a 42,66 ± 1,60 cm) (p < 0,001) (Tabla 6). Al observar la apertura de forma particular, se observó un aumento independiente en cada paciente, que se estabilizó a partir de la cuarta sesión. Los mejores resultados se lograron en las sesiones iniciales (Figura 7). 

**Tabla 6:** Apertura oral medida en escala métrica en milímetros (mm)

Sesión	Media	Desviación estándar	p*
1	32,08	4,38	> 0,001
2	39,75	2,80	
3	37,41	3,20	
4	41,41	2,01	
5	41,41	1,69	
6	42,66	1,60	

*Análisis realizado mediante prueba ANOVA (análisis de varianza) de modelo mixto.

## DISCUSIÓN

La fotobiomodulación es una terapia alternativa favorable para el tratamiento del dolor agudo a nivel de la ATM dados sus efectos bioestimulantes, ya que aumentan el metabolismo celular, lo cual promueve la oxidación de las mitocondrias al elevar la producción de ATP ^8^^,^^14^^,^^15^. Asimismo, su efecto analgésico fomenta la reducción del dolor agudo y crónico a través de un bloqueo de conducción y una alteración de los nociceptores A-delta y C, con acción a nivel del sistema nervioso central a través de transmisión ascendente y descendente ^12^^,^^15^.

La fotobiomodulación podría actuar inhibiendo la prostaglandina E2, así como promoviendo un mecanismo de liberación de endorfinas b mediante la estimulación de producción de ATP ^17^. De Freitas *et al.*^18^ demostraron, mediante un estudio en roedores, que el umbral nociceptivo a nivel de la ATM viene dado por la sustancia P, el péptido relacionado con el gen de la calcitonina (CGRP) y el receptor de potencial transitorio V1 (TRPV-1). Estos tienen efecto sobre la actividad excitatoria neuronal, lo que promueve la transmisión de la información nociceptiva. Al aplicar la terapia de fotobiomodulación, se observó una reversión del estado hipernociceptivo en paralelo con una reducción en la expresión proteica de estos receptores.

La mayor controversia relacionada con la fotobiomodulación a nivel de la ATM se refiere a la dosis adecuada. Según algunos autores, esta falta de consenso generó muchos resultados controvertidos y difirió de la más amplia aceptación de protocolos láser para muchas condiciones clínicas ^9^^,^^10^^,^^17^^,^^19^.

La eficacia de la fotobiomodulación para el control del dolor agudo de los TTM depende de la fluencia, la potencia y la dosimetría. La terapia de fotobiomodulación requieren fluencias entre 0,05 y 10 J/cm^2 (^^10^^,^^18^. Los parámetros que determinan los efectos clínicos más evidentes están en el rango de fluencias de 1-10 J/cm^2^, pero los valores entre 1 y 5 J/cm^2^ y 10 J/cm^2^ son también aceptables ^13^. Diferentes estudios ^8^^,^^10^^,^^20^^,^^21^ han reportado dosis aplicadas hasta 100 J/cm^2^; sin embargo, fluencias hasta 30 J/cm^2^ en el área muscular y 12 J/cm^2^ en zona articular son suficientes para la disminución del dolor y un aumento significativo de la apertura oral.

Otros parámetros por evaluar, debido a la absorción y difusión de la luz en los tejidos, son las longitudes de onda y del tipo de tejido que se irradia. La “ventana terapéutica” en el espectro de rango de longitudes de onda útiles para este tipo de aplicación se encuentra entre 600 y 1150 nm ^10^^,^^12^^,^^20^. El uso de la longitud de onda dual 810/980 nm logró obtener una absorción adecuada en los tejidos tanto articulares como musculares.

Khalighi *et al*. ^22^ realizaron un estudio controlado aleatorizado doble ciego en el que incluyeron a 24 pacientes y les aplicaron 12 sesiones de irradiación con una longitud de onda de 810 nm en modo continuo, utilizando un diámetro de 9 mm con una potencia comprendida entre 0,2 W y 0,5 W. Se evaluaron como parámetros el nivel del dolor mediante la escala EVA y la apertura bucal máxima sin dolor. Como resultado, se obtuvo una disminución del dolor y un aumento de la apertura bucal máxima, aunque en el aspecto estadístico no se hallaron cambios significativos.

Monteiro *et al*. ^23^ realizaron a cabo un ensayo clínico, aleatorizado y controlado con placebo en 42 pacientes con dolor. Se asignaron 2 grupos de manera aleatoria un grupo de intervención sometido a la aplicación de una longitud de onda de 635 nm láser de diodo, utilizando una pieza de mano de 8 mm de diámetro en modo de contacto. Se usaron 8 J/cm^2^ durante un período de 20 segundos aplicado sobre puntos sensibles donde los participantes informaron dolor y un grupo de placebo con el mismo protocolo, pero sin activación láser. Todos los pacientes recibieron cuatro sesiones de tratamiento durante 4 semanas. Se evaluó la percepción personal del dolor mediante escala EVA, apertura máxima de la boca sin dolor ni asistencia, y sensibilidad provocada durante la palpación muscular. Se utilizó como resultado primario, evaluada al inicio y en el seguimiento por evaluadores ciegos y calibrados, quienes concluyeron que hubo una mejoría de los síntomas dolorosos de los TTM, lo cual aumentó la apertura de la boca y mejoró los aspectos relacionados con la masticación. Ellos sugieren que, para confirmar la reproducibilidad de sus datos, se debe realizar la fotobiomodulación junto con otras formas de intervención.

Carvalho *et al*. ^24^ propusieron utilizar una combinación de diferentes longitudes de onda: 660 nm y/o 780 nm, 790 nm o 830 nm, argumentando que la asociación de rojo e infrarrojo con la luz láser podría ser eficaz para reducir el dolor en los trastornos temporomandibulares. Maracci *et al*. ^25^ realizaron un ensayo clínico aleatorizado doble ciego donde compararon el uso de férulas oclusales y la terapia de láser de baja frecuencia en 384 pacientes. Los parámetros del dispositivo láser utilizado fueron longitud de onda = 808 nm (infrarrojos), potencia de 100 mW, fluencia de 80 J/cm^2^, 22 s por aplicación y una distancia de al menos al menos 1 cm entre cada sitio. Los resultados obtenidos fueron beneficiosos para ambos grupos, pero cabe destacar que el grupo de fotobiomodulación tuvo una disminución del dolor de manera más rápida que el grupo en donde se utilizaron férulas oclusales. Un aspecto importante descrito en el estudio anterior es la terapia coadyuvante de la fotobiomodulación en conjunto con las diferentes terapias descritas en la literatura ^8^^,^^10^^,^^13^^,^^25^, ya que el uso de férulas oclusales debe continuarse tras la aplicación del láser de baja frecuencia.

Finalmente, es importante destacar las limitaciones del uso de la fotobiomodulación porque, aunque puede aliviar el dolor en los TTM a corto plazo, no debe ser considerada una terapia definitiva. La fotobiomodulación permite el manejo del dolor agudo, sin embargo, sus efectos no son curativos, por lo que los pacientes requieren de un adecuado tratamiento e identificación de las causas asociadas a cada trastorno de forma individual para un tratamiento que resuelva la sintomatología de forma sostenible.

## LIMITACIONES

Los estudios de reporte de casos, aunque valiosos para generar hipótesis, se basan en un pequeño número de individuos, por lo que no se pueden extrapolar a poblaciones más grandes. Asimismo, la ausencia de un grupo control dificulta determinar si el tratamiento estudiado, en este caso la terapia láser, es la causa del resultado. Por tal razón, el diseño de reporte de caso usado en esta investigación no permite establecer conclusiones definitivas sobre la eficacia del tratamiento, lo cual genera la necesidad de realizar estudios de evidencia científica más elevados como los ensayos clínicos aleatorizados. 

## CONCLUSIÓN

Los resultados del presente estudio confirman que la fotobiomodulación es eficaz para reducir el dolor agudo relacionado con la ATM. También destaca que el uso de láser diodo dual 810-980 nm es una alternativa no invasiva, con pocos efectos secundarios, que puede ser beneficiosa y tener resultados positivos.

## Financiamiento:

Autofinanciado
